# Supplementary material for: Unveiling the Bioactive Potential of the Invasive Jellyfish Phyllorhiza punctata Through Integrative Transcriptomic and Proteomic Analyses
Source: Biomolecules. 2025 Aug 4;15(8):1121. doi: 10.3390/biom15081121 (PMC12383608; doi:10.3390/biom15081121)
Supplement: Supplementary file 1 [file biomolecules-15-01121-s001.zip › Suplementary Material/FileS3/PEPFOLD4/XP_065058123_5088/clusters.html]

 PEP-FOLD clusters


Download archive of all models  here   

|  |  |  |
| --- | --- | --- |
| # | file | sOPEP |
| # cluster 1 - Size: 51 |  |  |
|  | XP\_065058123\_5088-model1 | -19.4695 |
|  | XP\_065058123\_5088-model1.1 | -19.4695 |
|  | XP\_065058123\_5088-model1.2 | -19.3591 |
|  | XP\_065058123\_5088-model1.3 | -19.3591 |
|  | XP\_065058123\_5088-model1.4 | -19.3591 |
|  | XP\_065058123\_5088-model1.5 | -19.3591 |
|  | XP\_065058123\_5088-model1.6 | -19.3591 |
|  | XP\_065058123\_5088-model1.7 | -19.3591 |
|  | XP\_065058123\_5088-model1.8 | -19.3591 |
|  | XP\_065058123\_5088-model1.9 | -19.3591 |
|  | XP\_065058123\_5088-model1.10 | -19.2852 |
|  | XP\_065058123\_5088-model1.11 | -19.2852 |
|  | XP\_065058123\_5088-model1.12 | -19.2852 |
|  | XP\_065058123\_5088-model1.13 | -19.2852 |
|  | XP\_065058123\_5088-model1.14 | -19.2508 |
|  | XP\_065058123\_5088-model1.15 | -19.2461 |
|  | XP\_065058123\_5088-model1.16 | -19.2461 |
|  | XP\_065058123\_5088-model1.17 | -19.2461 |
|  | XP\_065058123\_5088-model1.18 | -19.1761 |
|  | XP\_065058123\_5088-model1.19 | -19.1744 |
|  | XP\_065058123\_5088-model1.20 | -19.1744 |
|  | XP\_065058123\_5088-model1.21 | -19.1744 |
|  | XP\_065058123\_5088-model1.22 | -19.1744 |
|  | XP\_065058123\_5088-model1.23 | -19.1744 |
|  | XP\_065058123\_5088-model1.24 | -19.1744 |
|  | XP\_065058123\_5088-model1.25 | -19.1744 |
|  | XP\_065058123\_5088-model1.26 | -19.1744 |
|  | XP\_065058123\_5088-model1.27 | -19.1744 |
|  | XP\_065058123\_5088-model1.28 | -19.1744 |
|  | XP\_065058123\_5088-model1.29 | -19.1744 |
|  | XP\_065058123\_5088-model1.30 | -19.1744 |
|  | XP\_065058123\_5088-model1.31 | -19.1744 |
|  | XP\_065058123\_5088-model1.32 | -19.1744 |
|  | XP\_065058123\_5088-model1.33 | -19.1744 |
|  | XP\_065058123\_5088-model1.34 | -19.1744 |
|  | XP\_065058123\_5088-model1.35 | -19.1744 |
|  | XP\_065058123\_5088-model1.36 | -19.1744 |
|  | XP\_065058123\_5088-model1.37 | -19.1744 |
|  | XP\_065058123\_5088-model1.38 | -19.1744 |
|  | XP\_065058123\_5088-model1.39 | -19.1744 |
|  | XP\_065058123\_5088-model1.40 | -19.1744 |
|  | XP\_065058123\_5088-model1.41 | -19.1744 |
|  | XP\_065058123\_5088-model1.42 | -19.1744 |
|  | XP\_065058123\_5088-model1.43 | -19.1744 |
|  | XP\_065058123\_5088-model1.44 | -19.1744 |
|  | XP\_065058123\_5088-model1.45 | -19.136 |
|  | XP\_065058123\_5088-model1.46 | -19.136 |
|  | XP\_065058123\_5088-model1.47 | -19.136 |
|  | XP\_065058123\_5088-model1.48 | -19.136 |
|  | XP\_065058123\_5088-model1.49 | -19.1354 |
|  | XP\_065058123\_5088-model1.50 | -19.1354 |
| # cluster 2 - Size: 10 |  |  |
|  | XP\_065058123\_5088-model2 | -19.2869 |
|  | XP\_065058123\_5088-model2.1 | -19.2869 |
|  | XP\_065058123\_5088-model2.2 | -19.2427 |
|  | XP\_065058123\_5088-model2.3 | -19.1725 |
|  | XP\_065058123\_5088-model2.4 | -19.1725 |
|  | XP\_065058123\_5088-model2.5 | -19.1363 |
|  | XP\_065058123\_5088-model2.6 | -19.1363 |
|  | XP\_065058123\_5088-model2.7 | -19.1293 |
|  | XP\_065058123\_5088-model2.8 | -19.0742 |
|  | XP\_065058123\_5088-model2.9 | -19.0666 |
| # cluster 3 - Size: 31 |  |  |
|  | XP\_065058123\_5088-model3 | -19.2856 |
|  | XP\_065058123\_5088-model3.1 | -19.2856 |
|  | XP\_065058123\_5088-model3.2 | -19.2856 |
|  | XP\_065058123\_5088-model3.3 | -19.173 |
|  | XP\_065058123\_5088-model3.4 | -19.173 |
|  | XP\_065058123\_5088-model3.5 | -19.173 |
|  | XP\_065058123\_5088-model3.6 | -19.173 |
|  | XP\_065058123\_5088-model3.7 | -19.173 |
|  | XP\_065058123\_5088-model3.8 | -19.173 |
|  | XP\_065058123\_5088-model3.9 | -19.173 |
|  | XP\_065058123\_5088-model3.10 | -19.173 |
|  | XP\_065058123\_5088-model3.11 | -19.173 |
|  | XP\_065058123\_5088-model3.12 | -19.1362 |
|  | XP\_065058123\_5088-model3.13 | -19.1362 |
|  | XP\_065058123\_5088-model3.14 | -19.1362 |
|  | XP\_065058123\_5088-model3.15 | -19.1362 |
|  | XP\_065058123\_5088-model3.16 | -19.1362 |
|  | XP\_065058123\_5088-model3.17 | -19.1362 |
|  | XP\_065058123\_5088-model3.18 | -19.1362 |
|  | XP\_065058123\_5088-model3.19 | -19.1362 |
|  | XP\_065058123\_5088-model3.20 | -19.1362 |
|  | XP\_065058123\_5088-model3.21 | -19.1334 |
|  | XP\_065058123\_5088-model3.22 | -19.1164 |
|  | XP\_065058123\_5088-model3.23 | -19.1007 |
|  | XP\_065058123\_5088-model3.24 | -19.1007 |
|  | XP\_065058123\_5088-model3.25 | -19.0945 |
|  | XP\_065058123\_5088-model3.26 | -19.0449 |
|  | XP\_065058123\_5088-model3.27 | -19.0246 |
|  | XP\_065058123\_5088-model3.28 | -19.0232 |
|  | XP\_065058123\_5088-model3.29 | -19.0232 |
|  | XP\_065058123\_5088-model3.30 | -19.0216 |
| # cluster 4 - Size: 5 |  |  |
|  | XP\_065058123\_5088-model4 | -19.2593 |
|  | XP\_065058123\_5088-model4.1 | -19.2593 |
|  | XP\_065058123\_5088-model4.2 | -19.1081 |
|  | XP\_065058123\_5088-model4.3 | -19.1081 |
|  | XP\_065058123\_5088-model4.4 | -19.1081 |
| # cluster 5 - Size: 1 |  |  |
|  | XP\_065058123\_5088-model5 | -19.1816 |
| # cluster 6 - Size: 2 |  |  |
|  | XP\_065058123\_5088-model6 | -19.0849 |
|  | XP\_065058123\_5088-model6.1 | -19.0849 |
|  |  |  |
